# The effect of additional carbohydrate supplements for 7 days after prolonged interval exercise on exercise performance and energy metabolism during submaximal exercise in team-sports athletes

**DOI:** 10.20463/jenb.2018.0005

**Published:** 2018-03-31

**Authors:** Hun-Young Park, Jisu Kim, Miyoung Park, Nana Chung, Kiwon Lim

**Affiliations:** 1. Physical Activity and Performance Institute, Konkuk University, Seoul Republic of Korea; 2. Laboratory of Exercise Nutrition, Department of Physical Education, Konkuk University, Seoul Republic of Korea

**Keywords:** Team-sports athletes, carbohydrate supplements, exercise performance, energy metabolism during submaximal exercise

## Abstract

**[Purpose]:**

The purpose of our study was to determine the effectiveness of carbohydrate loading by additional carbohydrate supplements for 7 days after prolonged interval exercise on exercise performance and energy metabolism during submaximal exercise in team-sports athletes.

**[Methods]:**

Twenty male team-sports athletes (14 soccer and 6 rugby players) volunteered to participate in the study and were equally divided into the experimental group (EXP, *n*=10) performing additional carbohydrate supplementation for 7 days after prolonged interval exercise until blood glucose level reaches 50 mg/dL or less and the control group (CON, *n*=10). Then, maximal oxygen consumption (VO_2max_) and minute ventilation (VE), oxygen consumption (VO_2_), carbon dioxide excretion (VCO_2_), respiratory exchange ratio (RER), blood glucose level, and blood lactate level were measured in all team-sports players during submaximal exercise corresponding to 70% VO2max before and after intervention.

**[Results]:**

There was no significant interaction in all parameters, but team-sports players in the EXP presented more improved VO2max (CON vs EXP = vs 5.3% vs 6.3%), VE (CON vs EXP = vs 3.8% vs 6.6%), VO2 (CON vs EXP = vs 8.5% vs 9.9%), VCO2 (CON vs EXP = vs 2.8% vs 4.0%), blood glucose level (CON vs EXP = vs -12.9% vs -7.6%), and blood lactate level (CON vs EXP = -18.2% vs -25%) compared to those in the CON.

**[Conclusion]:**

These findings showed that additional carbohydrate supplementation conducted in our study is not effective in exercise performance and energy metabolism during submaximal exercise.

## INTRODUCTION

All athletes are part of teams whether as track athletes, swimmers, or football players; however, team-sports athletes usually mean players who depend on the mutual cooperation of other players to score more goals/points than the opposition, such as in football, soccer, field and ice hockey, rugby, basketball, and lacrosse^1^. The common pattern of play in “team sports” is “stop and go,” i.e., where players perform repeated bouts of brief high-intensity exercise punctuated by lower-intensity activity; carbohydrate serves as a major energy source for the athletic performance of team-sports players^1-2^. In addition, the importance of carbohydrates increases with increasing exercise intensity, but because of the limited carbohydrate reserves in the body, the depletion of liver and intramuscular glycogen by high-intensity exercise for a long time is a determining factor in the athletic performance of team-sports players^3-4^. Carbohydrate depletion in the body by high-intensity team-sports performed over a long period of time impairs athletic performance due to inhibition of fat metabolism and accumulation of metabolites such as ammonia (NH3), lactate, hydrogen ion (H+), and inorganic phosphates (Pi)^5-7^.

Carbohydrate loading by high-carbohydrate diet or additional carbohydrate supplements after depletion of carbohydrate through prolonged high-intensity exercise increases the level of glycogen synthesis by increasing glucose transporter (GLUT)-4 and glycogen synthases concentrations^8-10^. In addition, carbohydrate loading improves the secretion of anabolic hormones (e.g., growth hormone [GH] and testosterone) and the efficiency of glucose by increased insulin secretion with increasing blood glucose concentration^11,12^. It also reduces free fatty acid (FFA) and glycerol levels in the blood5. Besides, additional carbohydrate supplements before exercise lead to an increase in carbohydrate metabolic efficiency by the activation of intramuscular pyruvatehydrogenase complex (PDC)^13^, thereby increasing intramuscular adenosine triphosphate (ATP), creatine phosphate (PCr), and glycogen content via increased excess post-exercise oxygen consumption (EPOC)^14^. These carbohydrate loading effects by a high-carbohydrate diet or additional carbohydrate supplements have been reported to maximize liver and muscle glycogen storage and improve exercise performance of various athletes^4,9,15,16^.

In various previous studies, Bussau et al.^9^ reported higher intramuscular glycogen content for 24 h in endurance-trained male cyclists or triathletes aged between 18 and 30 years after eating 10g∙day^-1^∙kg^-1^ body mass high-carbohydrate foods having a high glycemic index over 3 days, while remaining physically inactive. Sullo et al.^15^ found that male professional long-distance athletes with carbohydrate loading had better 25 km time trial performance and higher glucose and lactate concentrations in the last 5 km. Tarnopolsky et al.^16^ reported enhanced carbohydrate utilization capacity and exercise performance in seven endurance-trained male athletes after 4 days on a high-carbohydrate diet (55–60 to 75% of energy intake). Foskett et al.^5^ reported 6.4% CHO-E solution intake immediately before each trial (8 mL∙kg^-1^ BM) and at 15-min intervals (3mL∙kg^-1^ BM) during intermittent high-intensity running to fatigue performed after carbohydrate loading for 2 days improves endurance capacity in six recreationally active male games players with high pre-exercise muscle glycogen concentrations. Vandenbogaerde et al.^4^, based on the meta-analysis of 73 studies, verified that an adequate carbohydrate supplement intake of about 0.7 g∙kg^-1^∙hr^-1^ before and during exercise enhances the endurance performance by 2 to 6%. However, most previous studies have examined the effects of carbohydrate loading with diet or supplements in endurance athletes such as middle- and long-distance runners or cyclists, while studies of team-sports players who performed various types of prolonged high-intensity exercise are relatively inadequate and the results are also unclear^1^.

Therefore, the purpose of this study was to investigate the effect of additional carbohydrate supplements for 7 days after prolonged interval exercise on exercise performance and energy metabolism during submaximal exercise in team-sports athletes. We hypothesized that 7 days of additional carbohydrate supplements after prolonged interval exercise may enhance exercise performance with increased carbohydrate utilization capacity and carbohydrate-sparing effect with improved fat oxidation during submaximal exercise.

## METHODS

### Participants

Twenty team-sports athletes (14 soccer and 6 rugby players) who belong to the Korean Football Association and Korean Rugby Union volunteered to participate in the study. They were non-smokers and had no history of musculoskeletal, cardiovascular, or pulmonary disease. All participants received information about the purpose, process, and possible adverse effects of the study and provided written consent prior to the start of the study. There were no significant differences in physical characteristics among group (Table 1). All procedures were in accordance with the ethical standards of the responsible committee on human experimentation and Declaration of Helsinki.

**Table 1.** Participant characteristics

Variables	Control group	Experimental group
Number (N)	10	10
Age (years)	24.2±2.6	24.0±3.3
Height (cm)	180.2±4.8	178.2±4.9
Weight (kg)	74.6±7.7	72.1±5.8
Body fat mass (kg)	59.9±5.2	57.8±5.4
Body fat (%)	15.1±2.9	14.8±3.1

**Table 2.** Ingredients and nutrition of the carbohydrate supplements

Nutrients (per 100g)	Content	Nutritional value (%)
Calorie	382kcal	
Carbohydrate	96g	29%
Sugars	0g	
Protein	0g	
Lipid	0g	
Cholesterol	0mg	
Sodium	10mg	0.5%

*Note*. Nutritional value: percentage of daily value

### Experimental design

Twenty team-sports athletes were equally assigned to either the control group (CON = 10, 7 soccer and 3 rugby players) and experimental group (EXP = 10, 7 soccer and 3 rugby players)according to their physical characteristics (Table 1). Then, all participants were given appropriate intervention for 7 days for each group.

The CON was fed a normal meal for 7 days without any nutrients and calorie restriction. On the other hand, the EXP received an intervention for 7 days of carbohydrate loading. On the 1st day, intermittent exercise (1-min exercise and 30-s rest) corresponding to 90% maximal heart rate (HR max) in the treadmill was repeatedly carried out until the blood glucose level fell below 50 mg/dL in order to induce carbohydrate loading effect by the depletion of intramuscular glycogen, and normal diet was given without any nutrients and calorie restriction. On the 2nd and 4thday, after a normal meal, 50 g of additional carbohydrate supplements (Sollos-C Gold, Dowzon Pharm. Co., Korea) was provided 3 times daily. On the 5th and 7th days, the EXP consumed 100 g of additional carbohydrate supplements 3 times daily. The ingredients and nutrition of the carbohydrate supplements are shown in Table 2.

We designed a study to measure the effectiveness of additional carbohydrate supplements for 7 days after prolonged interval exercise on exercise performance and energy metabolism during submaximal exercise in team-sports athletes. Therefore, before and after intervention, all participants measured the maximal oxygen consumption (VO_2max_) via graded exercise test and energy metabolism at 30 min of continuous exercise on a treadmill with an intensity corresponding to 70% VO_2max_. Energy metabolism parameters during submaximal exercise were measured minute ventilation (VE), oxygen consumption (VO_2_), carbon dioxide excretion (VCO_2_), respiratory exchange ratio (RER), blood glucose level, and blood lactate level.

### Measurements

#### Height

Height was measured as the distance between the bottom of the foot and top of the head using an extensometer (PKS-1008, Japan).

#### Body composition

Body weight, free fat mass, body fat mass, and percent of body fat were measured using an InBody770 (InBody, Korea). All participants fasted overnight prior to measurement of body composition. They wore lightweight clothing and were asked to remove any metal items.

#### Exercise performance

To evaluate exercise performance, VO_2max_ was measured pre and post intervention using the modified Bruce protocol for graded exercise testing on a treadmill (S25T, STEX, Korea) with a breath-by-breath auto metabolism analyzer (Quark CPET, COSMED, Italy). The graded exercise test started at 2.7 km∙hr^-1^ and 0% inclination and increased by 1.3 to 1.4 km∙hr^-1^ and 2% inclination every 3 min until voluntary exhaustion. The athlete is considered to have reached their VO_2max_ if several of the following occurred: a plateau or “peaking over” in oxygen uptake, reaching the maximal heart rate, attainment of a respiratory exchange ratio of 1.15 or greater, and volitional exhaustion.

#### Energy metabolism during submaximal exercise

Energy metabolic parameters were measured over the 30 min of submaximal exercise. VE, VO_2_, VCO_2_, and RER were analyzed at every minute during rest and submaximal exercise using the breath-by-breath auto metabolism analyzer (Quark CPET, COSMED, Italy), a treadmill (S25T, STEX, Korea), and a breathing valve in the facemask form. Blood glucose and lactate levels were analyzed at rest, 5, 10, 20, and 30 min using the YSI2300 Stat Plus (YSI Corp., USA). To measure the blood glucose and lactate level, we collected 80 uL of blood in a capillary tube using the fingertip method. The summation value was used as a measure of VE, VO_2_, and VCO_2_. The average value was used as measurement value in RER, blood glucose level, and blood lactate level.

#### Statistical analysis

Means and standard deviations (SD) were calculated for each primary dependent variable. Normality of distribution of all outcome variables were verified using the Kolmogorov-Smirnov test. A two-way analysis (time × group) of variance with repeated measures of the “time” factor was used to evaluate the effects of training programs on each dependent variable. If a significant interaction effect or main effect within the time was found, a Bonferroni posthoc test was used to identify within-group changes over time. Additionally, the paired t-test was used to compare post- versus pre-intervention values of dependent variables in each group separately. The level of significance was set a priori at 0.05.

## RESULTS

### Exercise performance

Pre- and post-intervention data for exercise performance in both groups are shown in Table 3. No significant interaction was observed in VO_2max_, but significant main effects within time were observed (F = 5.086, *p* = 0.037, *η*^2^ = 0.220).In the change rate via intervention in each group, team-sports players in the EXP showed more increased tendency in VO_2max_(CON vs EXP = 5.3% vs 6.3%) compared to those in the CON.

**Table 3.** Change of VO2max(mL∙kg^-1^∙min^-1^) by additional carbohydrate supplementsfor 7 days after prolonged interval exercise

Group	Pre-test	Post-test	△%	ANOVA F-value	
CON	58.2±5.8	61.3±7.2	5.3%	Time	5.086*
				Group	0.336
EXP	56.8±4.9	60.4±3.1	6.3%	Time × Group	0.028

*Note*. VO_2max_, maximal oxygen consumption; CON, control group; EXP, experimental group

* *p*<0.05: significant main effect within the intervention

### Energy metabolism during submaximal exercise

Table 4 depicts pre- and post-test data for energy metabolism during submaximal exercise in both groups. There was no significant interaction in all variables (VE, VO2, VCO2, RER, blood glucose level, and blood lactate level), but significant main effects within time were observed in VE (F = 11.348, *p* = 0.003, *η*^2^ = 0.387), VO2 (F = 15.616, *p* = 0.001, *η*^2^ = 0.465), VCO2 (F = 5.689, p = 0.028, *η*^2^ = 0.240), RER (F = 13.145, *p* = 0.002, *η*^2^ = 0.422), blood glucose level (F = 25.016, *p* = 0.000, *η*^2^ = 0.582), and blood lactate level (F = 16.089, *p* = 0.001, *η*^2^ = 0.472). In the change rate via intervention in each group, team-sports players in the EXP presented more improved tendency in VE (CON vs EXP = vs 3.8% vs 6.6%), VO2 (CON vs EXP = vs 8.5% vs 9.9%), VCO2 (CON vs EXP = vs 2.8% vs 4.0%), blood glucose level (CON vs EXP = -12.9% vs -7.6%), and blood lactate level (CON vs EXP = -18.2% vs -25%) during submaximal exercise compared to those in the CON.

**Table 4.**
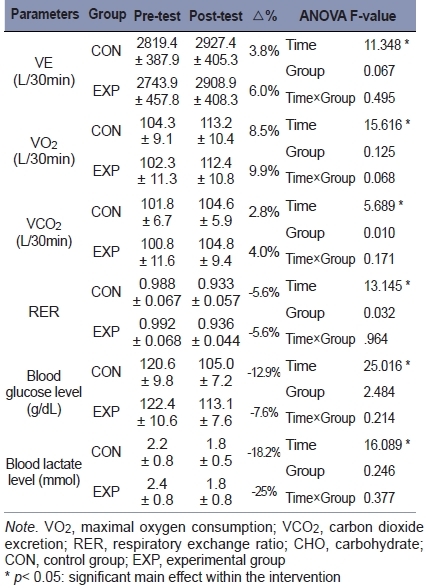
Change of energy metabolism parameters during submaximal exercise by additional carbohydrate supplements for 7 days after prolonged interval exercisee

## DISCUSSION

Carbohydrate loading by a high-carbohydrate diet or additional carbohydrate supplements after depletion of carbohydrate through prolonged high-intensity exercise have been reported to increase intramuscular glycogen storage and thereby improve endurance and athletic performance^11,17-18^. In this study, there was no significant interaction in all parameters related to exercise performance (VO_2max_) and energy metabolism (VE, VO_2_, VCO_2_, RER, blood glucose level, and blood lactate level) during submaximal exercise in team-sports athletes. These results show that additional carbohydrate supplements for 7 days after prolonged interval exercise are not effective for improving exercise performance in team-sports athletes such as soccer and rugby players. However, more improvement in the EXP was observed in the VO2max, blood glucose level, and blood lactate level compared to those in the CON. These results presented the possibility that additional carbohydrate supplements for 7 days after prolonged interval exercise performed in this study may be effective.

The main finding of this study was that there was no significant difference in VO2max among the two intervention groups. Although the EXP showed a larger increase rate (CON vs EXP = 5.3% vs 6.3%) than the CON, this difference did not result in a significant interaction. A few previous studies have reported that carbohydrate loading by a high-carbohydrate diet or additional carbohydrate supplements after depletion of carbohydrate does not affect exercise performance^16,19^. Andrews et al.^16^ reported that despite the increase in carbohydrate oxidation, the 24.2-km time trial was not improved in 8 female aerobic-trained athletes after 4 days on a high carbohydrate diet (75% of energy intake).Tarnopolsky et al.^19^ reported that muscle glycogen concentration did not increase by 0% in response to dietary carbohydrate from 55–60 to 75% of energy intake for a period of 4 days (carbohydrate loading) and there was no corresponding increase in performance time during an 85% VO2peak trial (5%) in 8 endurance-trained female athletes. However, many previous studies revealed that carbohydrate loading is effective in the enhancement of exercise performance. Walker et al.^20^ reported that aerobic-trained women increased muscle glycogen content in response to a high-carbohydrate diet (75% of energy intake) for a period of 4 days. Moreover, the increase in muscle glycogen, and possibly liver glycogen, after a high-carbohydrate diet was associated with increased cycling performance to volitional exhaustion at approximately 80% VO_2max_. Foskett et al.^5^ proved that after consumption of a carbohydrate-rich diet, carbohydrate supplementation (90 g∙h^-1^) in the form of a 6.4% carbohydrate-electrolyte solution during exercise led to an improvement in high intermittent running capacity, but it did not affect muscle glycogen use. In our study, we presumed that the positive results of exercise performance via carbohydrate loading by a high-carbohydrate diet or additional carbohydrate supplements after depletion of carbohydrate through prolonged high-intensity exercise were not observed for the following reasons: First, we did not use the method of high-carbohydrate diet intake which corresponds to 75–80% based on the analysis of the dietary intake which is obtained through accurate dietary investigation. After meals, to emphasize practical aspects, 50 g (2nd to 4th days) and 100 g (4th to 7th days) of additional carbohydrate supplements without an accurate dietary investigation were provided in team-sports athletes 3 times daily. Second, unlike previous studies, we selected team-sports athletes rather than endurance athletes as subjects; therefore, athletic performance was evaluated by using VO_2max_ instead of exercise duration until exhaustion using a treadmill.

In addition, we measured energy metabolism through VE, VO_2_, VCO_2_, RER, blood glucose level, and blood lactate level over 30 min of submaximal exercise. As a result, all energy metabolic parameters showed no significant interaction. Thus, additional carbohydrate supplements performed in our study were not effective in energy metabolism during submaximal exercise. However, in the other variables except for RER, the change rate in the EXP showed a more positive tendency than that in the CON. In the case of previous studies related to energy metabolism, Tarnopolsky et al.^16^ reported a 41% increase in muscle glycogen as a result of carbohydrate loading by high-carbohydrate diets equivalent to 75% of energy intake for 4 days in 7 male endurance athletes. Andrew et al.^19^ reported that carbohydrate loading for 4 days in 8 aerobic-trained women showed a significantly higher RER and blood lactate level and lower blood glycerol concentration. Foskett et al.^5^ announced that after 2 days of carbohydrate loading, carbohydrate-electrolyte solution intake at rest (8 mL∙kg^-1^) and during interval exercise (3 mL∙kg^-1^)resulted in higher blood insulin, glucose, and lactate levels and lower blood glycerol and free fatty acid concentration during exercise and recovery compared to those in the placebo group. In our study, the EXP showed an increased VE, VO_2_, and VCO_2_ during submaximal exercise compared to the CON. Based on the RER result, considering the same rate of change in carbohydrate and lipid oxidation between both groups during submaximal exercise, the increased relative exercise intensity (e.g., VO_2max_) in the EXP is considered to be caused by these results. Unlike Andrews et al.^19^ and Foskett et al.^5^, our study failed to identify higher RER, blood glucose level, and blood lactate level in the EXP compared to those in the CON; these results mean that our carbohydrate loading method does not lead to efficient carbohydrate loading effect. In other words, for team-sports athletes to have an efficient carbohydrate loading effect, we consider that it is desirable to take quantified carbohydrates from 75 to 80% of the daily energy intake for 4 to 7 days after the carbohydrate depletion by prolonged high-intensity interval exercise, and this carbohydrate loading method may increase the amount of intramuscular glycogen storage and improve energy metabolism and performance during exercise in team-sports athletes.

## CONCLUSION

Our results suggested that carbohydrate loading performed in this study had no effect on energy metabolism during submaximal exercise and exercise performance in team-sports athletes. These results are because teamsports athletes were not given the same dietary treatment control except for carbohydrate supplementation and carbohydrate supplements are used instead of quantified diets for supplementation.
